# Partial Cell Fate Transitions to Promote Cardiac Regeneration

**DOI:** 10.3390/cells13232002

**Published:** 2024-12-04

**Authors:** Jianchang Yang

**Affiliations:** Michael E. DeBakey Department of Surgery, Baylor College of Medicine, One Baylor Plaza, Houston, TX 77030, USA; jianchay@bcm.edu

**Keywords:** cardiac fibroblast, dedifferentiation, pluripotency, partial reprogramming, rejuvenation, Sall4, Gata4

## Abstract

Heart disease, including myocardial infarction (MI), remains a leading cause of morbidity and mortality worldwide, necessitating the development of more effective regenerative therapies. Direct reprogramming of cardiomyocyte-like cells from resident fibroblasts offers a promising avenue for myocardial regeneration, but its efficiency and consistency in generating functional cardiomyocytes remain limited. Alternatively, reprogramming induced cardiac progenitor cells (iCPCs) could generate essential cardiac lineages, but existing methods often involve complex procedures. These limitations underscore the need for advanced mechanistic insights and refined reprogramming strategies to improve reparative outcomes in the heart. Partial cellular fate transitions, while still a relatively less well-defined area and primarily explored in longevity and neurobiology, hold remarkable promise for cardiac repair. It enables the reprogramming or rejuvenation of resident cardiac cells into a stem or progenitor-like state with enhanced cardiogenic potential, generating the reparative lineages necessary for comprehensive myocardial recovery while reducing safety risks. As an emerging strategy, partial cellular fate transitions play a pivotal role in reversing myocardial infarction damage and offer substantial potential for therapeutic innovation. This review will summarize current advances in these areas, including recent findings involving two transcription factors that critically regulate stemness and cardiogenesis. It will also explore considerations for further refining these approaches to enhance their therapeutic potential and safety.

## 1. Clinical Challenges in Myocardial Regeneration

Heart diseases continue to be a major cause of global morbidity and mortality, with heart failure and myocardial infarction (MI) being the most significant contributors. Myocardial infarction, commonly known as a heart attack, occurs due to a blockage in the coronary arteries, leading to ischemia and the death of cardiomyocytes. If left untreated, the damaged myocardium is progressively replaced by fibrotic scar tissue, eventually leading to congestive heart failure (CHF), characterized by decreased contractile function and further deterioration of overall heart performance [1,2,3,4,5]. Despite advancements in medical management, the prognosis for CHF remains poor, with a 5-year survival rate of only 50%. Current treatments primarily focus on improving heart function through medications that reduce cardiac workload and prevent further damage, such as angiotensin-converting enzyme (ACE) inhibitors, beta-adrenergic blockers, mechanical devices (e.g., ventricular assistance devices and pacemakers), and surgical interventions (e.g., heart transplantation). However, these approaches mainly manage symptoms rather than restore the lost function of damaged cardiac tissue. They are also limited by low recovery efficacy, serious complications, and the global scarcity of donor hearts for transplantation [6,7,8,9]. A significant challenge is that the adult human heart has very limited regenerative capacity after cell loss, hampering the recovery of myocardial function after injury. To reverse the loss of myocytes and promote myocardial regeneration, cell transplantation has been a major area of research over the past two decades. Strategies have been developed to administer exogenous stem or reserve cells, such as bone marrow stem cells, mesenchymal stromal cells, skeletal muscle cells, cardiac progenitors, or pluripotent stem cells, into the infarct zone to enhance cardiac repair and functional recovery. However, clinical trials involving stem cell transplantation have encountered significant obstacles and inconsistent outcomes, such as inadequate implant phenotypes, poor survival, limited engraftment of transplanted cells into the host myocardium, or the risk of cardiac arrhythmias [10,11,12,13,14,15,16]. These limitations underscore ongoing challenges in the field and highlight the need for a deeper understanding of underlying mechanisms and innovative strategies to enhance therapeutic efficacy and reliability.

## 2. The Promise of Cardiac Cellular Reprogramming

The Nobel Prize-winning discovery of induced pluripotent stem cell (iPSC) technology in 2006, along with subsequent findings that iPSCs could be re-differentiated into cardiomyocyte-like cells, soon led to the discovery that specialized reprogramming factors (e.g., Gata4, Mef2C, and Tbx5) could be administered to cardiac fibroblasts to directly transdifferentiate them into “induced cardiomyocyte-like” (iCM) cells without going through a pluripotency stage [17,18,19]. Cardiac cellular transdifferentiation thus offers the potential to regenerate functional iCMs from cardiac fibroblasts in situ, turning scar tissue into functional myocardium while overcoming the challenges posed by exogenous cell delivery strategies. Studies, including our own, have shown that administering reprogramming factors can increase ventricular ejection fraction by up to 30% in both acute and chronic animal models of myocardial infarction, while reducing infarct size by nearly half [20,21,22,23,24]. These findings support the premise that direct cellular reprogramming is a promising new treatment for cardiomyopathy. Despite these encouraging results, however, it was soon revealed that cardiac fibroblasts from higher-order species (e.g., humans and pigs) are more resistant to trans-differentiation than rodent cells, likely due to more complex epigenetic barriers to reprogramming [25,26,27,28,29,30,31,32]. Expanded reprogramming cocktails have been explored to overcome this hurdle in human systems, incorporating additional transcription factors, microRNAs, small molecules, chemicals, growth factors, or p63 pathway inhibition [33,34,35,36,37]. While these approaches have improved outcomes to varying degrees, further in-depth exploration of innovative cocktails and mechanistic investigations is still necessary to fully realize their potential for clinical applications.

Another reprogramming approach involves the generation of induced cardiac progenitor cells (iCPCs). iCPCs are engineered from cardiac fibroblasts to acquire unlimited self-renewal capacity and cardiac potency, enabling them to differentiate into all three cardiac lineages: cardiomyocytes, endothelial cells, and smooth muscle cells. iCPCs do not typically express key pluripotency genes such as Oct4 and Nanog but are characterized by their expression of heart-specific multipotent progenitor markers such as Nkx2.5, Isl1, Flk1, and Gata4. As a result, iCPCs provide several benefits compared to iCMs or iPSCs, including their capacity to regenerate key cardiac lineages required for myocardial repair, a reduced risk of tumorigenesis, and the potential for autologous transplantation with minimized immune rejection [38,39,40,41,42]. Despite these benefits, the iCPC approaches often involve complex reprogramming factors and induction procedures that can hinder their clinical application. Current methods utilize up to 11 or 5 cardiac factors along with JAK/STAT signaling mediators [43]; multiple pluripotency regulators with refined chemical conditions or small molecules [44,45]; Ets2 and Mesp1 with TGF-β pathway members [46]; or CRISPR activation of endogenous genes including *Gata4*, *Nkx2.5*, and *Tbx5* [47,48,49]. These studies collectively support the applicability and regenerative capacity of iCPCs both in vitro and in vivo. However, despite the theoretical feasibility of producing iCPCs in situ through the local delivery of the required reprogramming factors, few successful attempts have been documented to date. This is likely due to challenges in delivering multiple factors and managing iCPC induction and progression within the myocardial microenvironment. Thus, there is a pressing need for refined reprogramming cocktails and improved induction strategies to advance this approach [50,51,52].

## 3. Induced Partial Cellular Fate Transitions and Recent Advances

As an additional strategy, the induction of cell fate transitions into a partially reprogrammed state has gained increasing attention. In fact, partial cell fate transitions represent one of the most natural processes occurring in the body. In response to injury or stress, certain tissue cells can partially revert to a more primitive state, regaining characteristics associated with stem cells, such as self-renewal and multipotency. These features promote reparative regeneration, rejuvenation, and healing while minimizing the risks of tumor formation. This adaptive response is essential for maintaining homeostasis and ensuring organism survival.

In induced partial reprogramming, many protocols have utilized time-controlled transient expression of Yamanaka factors (i.e., Oct4, Sox2, Klf4, and c-Myc; OSKM) to trigger this process without reaching full pluripotency. Additionally, several non-OSKM approaches are being explored. In comparison to the iCM and iCPC approaches, partial reprogramming presents some theoretical advantages for cardiac regeneration. By reverting to a plastic state, partially reprogrammed cells could adapt to the specific needs of injured heart tissue, aiding in the regeneration of damaged cell types. This can be driven by intrinsic mechanisms, such as retained epigenetic memory, which preserves lineage-specific regulatory frameworks conducive to repair [53], and dynamic cross-talk with local signaling within the tissue microenvironment, guiding context-dependent differentiation [54,55]. These intrinsic processes could help reduce risks like uncontrolled proliferation or inappropriate differentiation, presenting a potentially safer approach for restoring cardiac function.

Nevertheless, the pluripotency factor-induced mixed intermediate stem and progenitor states, along with the broad spectrum of acquired differentiation potentials, underscore that partial reprogramming remains a promising yet intricate and less well-defined concept. This complexity spans investigations involving diverse starting cell types, various pluripotency or multipotency induction protocols, growth signaling mediators, and different in vitro and in vivo models, making it challenging to precisely delineate the molecular and epigenetic modifications involved. To date, many OSKM-induced cardiac cell induction models have been explored, often in combination with small molecules, chemicals, or supportive culturing materials, as summarized below.

### 3.1. Partial Reprogramming of Cardiomyocytes In Vitro

The earliest efforts in partial (or “shortcut”) reprogramming emerged as researchers sought to adapt the principles of iPSC technology, directing the process toward cardiogenesis without reverting to full pluripotency. In 2011, Efe et al. transiently overexpressed OSKM in mouse embryonic fibroblasts (MEFs) and tail-tip fibroblasts (TTFs) to induce partially reprogrammed non-pluripotent colonies. These colonies were then exposed to defined media containing BMP4 and the JAK inhibitor JI1, which induced the cardiac progenitor cell (CPC) markers Nkx2.5, Gata4, and Flk1 and directed differentiation into spontaneously contracting patches of cardiomyocytes [56]. In another study, Wang et al. transiently overexpressed Oct4 alone, along with small molecules SB431542 (a TGFβ inhibitor), CHIR99021 (a GSK3β inhibitor), Parnate (an LSD1 inhibitor), and Forskolin (a cAMP activator) in MEFs and TTFs. This combination similarly induced contracting iCM clusters through an intermediate proliferative CPC stage, with CPCs also differentiating into endothelial cells and smooth muscle cells under specific conditions [57]. Furthermore, Fu et al. used an entirely chemical combination of CHIR99021, Forskolin, RepSox (an ALK5 inhibitor), valproic acid (an HDAC inhibitor), Parnate, and TTNPB (a retinoid activator) to reprogram these two fibroblast types, generating cardiomyocyte-like cells through a CPC stage without reaching full pluripotency [58]. In human foreskin fibroblasts (HFFs), Cao et al. identified a combination of nine small molecules that epigenetically activated cardiogenic gene programs, resulting in sequential expression of gene markers for mesoderm (*KDR*, *MESP1*, and *BRACHYURY*), second heart field progenitors (*ISL1*, *HAND2*, *MEF2C*, and *GATA4*), and cardiomyocytes (*TNNT2*, *MYH6*, and *NPPA*), ultimately producing chemically induced beating cardiomyocytes (ciCMs) [25]. Additionally, various supporting materials such as Matrigel, engineered polyethylene glycol (PEG) hydrogels, and 3D fibrin gels have been employed in these models to enhance reprogramming efficiency [59,60]. Studies have also been conducted in bone marrow-derived mesenchymal stromal cells (MSCs), where co-culturing with embryonic cardiomyocytes led to the expression of cardiac markers, such as Nkx2.5 and ANF, in the MSCs. Despite this, the MSCs retained their original stromal cell phenotype, rather than fully differentiating into cardiomyocytes [61].

In another study targeting cultured postnatal cardiomyocytes extracted from both rats and mice, the temporary expression of adenoviral OSKM induced a partially dedifferentiated state characterized by enhanced cell proliferation. This state was marked by the temporary downregulation of cardiomyocyte-specific markers (*cTNT*, *Myh6*, and *Myh7*), disassembly or absence of sarcomere structures, increased expression of dedifferentiation markers (*Dppa4* and *E-cadherin*), and the presence of proliferative Ki67+ cardiomyocytes. Notably, cardiomyocyte-specific morphology, gene expression, and contractile activity were spontaneously recovered by day 15 following viral transduction [62]. Supporting the partial reprogramming and rejuvenation concept, Chuang et al. demonstrated the feasibility of partially converting iPSC-derived cardiomyocytes into neurons by introducing the neurogenic transcription factors Brn2, Ascl1, Myt1l, and NeuroD. This approach generated a significant number of cells expressing markers of both cardiomyocytes and neurons, suggesting the presence of cells in an intermediate, partially reprogrammed state. The observation that only three or four neuronal transcriptional factors are needed to convert mesoderm-derived, electrophysiologically active CMs into ectoderm-derived, electrophysiologically active neuronal-like cells suggests that a small number of key transcription factors are sufficient to regulate distinct cell fates [63]. Taken together, these in vitro studies highlight a partially cardiogenic fate transition process stimulated by various factors in both human and rodent models. They provide valuable insights for mechanistic exploration, innovative protocols, and applications such as cardiac tissue engineering, drug screening, and organoid development. However, since the starting fibroblasts are not necessarily of cardiac origin, further investigations are needed to assess their in situ cardiac regenerative relevance and enhance their therapeutic potential.

### 3.2. In Vivo Models of Partial Reprogramming

In animal models, most in vivo partial reprogramming studies have centered on longevity research and rejuvenation, with the aim of epigenetically reversing markers of cellular aging and restoring youthful functionality. Many significant studies on this topic have been thoroughly reviewed elsewhere [64,65,66,67,68,69,70,71,72,73]. Additionally, tissue-targeted partial reprogramming strategies have been explored. For instance, Wang et al. reported that transient myofiber-specific OSKM expression using the *Acta1*-Cre system activated muscle stem cells (satellite cells, or SCs) in mice, which accelerated muscle regeneration. Mechanistically, OSKM expression in myofibers regulates genes crucial for the SC microenvironment, such as p21, which in turn downregulates the myofiber-secreted niche factor Wnt4 [74]. Hishida et al. developed regulatable hepatocyte-specific OSKM expression in *Alb*-Cre mice, which rapidly and transiently induces a proliferative, plastic progenitor state, resulting in enhanced liver regeneration [75]. Similarly, Kim et al. induced the in vivo partial dedifferentiation of intestinal epithelial cells using OSKM, revealing molecular changes akin to those observed during the intestinal regeneration process, which facilitated tissue regeneration following damage [76]. OSKM-induced partial reprogramming also reduced fibrosis and improved tissue healing in mouse excisional and incisional wound models by inhibiting fibroblast transdifferentiation to myofibroblasts [77]. In the neural context, transient reprogramming has demonstrated promise in several animal models of central nervous system (CNS) diseases, promoting the generation of new neurons, improving functional outcomes, and reducing scar formation [78,79,80,81,82,83,84]. Additionally, non-OSKM approaches have also been documented. Chandrakanthan et al. transiently applied a combination of 5-azacytidine and the growth factor PDGF-AB to mature bone and fat cells, inducing the formation of induced multipotent stem (iMS) cells that expressed low levels of pluripotency genes, including Oct4, Myc, Sox2, Klf4, and Nanog. These iMS cells demonstrated long-term self-renewal and serial clonogenicity and contributed to tissue regeneration in a context-dependent manner when introduced in vivo, without developing teratomas [85].

In the cardiac field, however, studies investigating in vivo partial reprogramming approaches remain very limited. Two notable studies have explored this area. Kisby et al. intramyocardially injected short-term adenoviral vectors encoding OSKM factors into both healthy and MI-injured mouse hearts. The expression of these factors induced the transient upregulation of several endogenous pluripotency genes (*endo-Oct3/4* and *Gdf3*) and reprogramming-related genes (*Cdh1* and *Fut4*), while markers of fully dedifferentiated cells, including *Nanog,* remained silenced, with no teratoma formation observed, consistent with a partially reprogrammed process. However, this strategy was unable to elicit a significant regenerative response in the injured myocardium, possibly due in part to the relatively low rate of vector transduction (an average of 11.8%) in the myocardium [86]. In comparison, in a separate study, Chen et al. employed temporally controlled, heart-specific OSKM expression in mice, which led to the dedifferentiation of adult cardiomyocytes and reentry into the cell cycle, with a gene expression profile resembling that of fetal cardiomyocytes. In MI-injured hearts, this approach notably decreased fibrotic scar size and improved ventricular ejection fraction, highlighting its potential therapeutic relevance. However, it is important to note that with long-term OSKM treatments, cardiomyocytes either entered an irreversible neonatal state that could not sustain function or progressed to a pluripotent state that led to teratoma formation, resulting in premature death [87]. This dose- and time-controlled pattern aligns with the partial reprogramming process and trajectory in somatic cells, further highlighting the need to carefully manage reprogramming processes and safety concerns in therapeutic settings.

## 4. Advancing Cardiac Partial Reprogramming Strategies

Building on the outcomes of previous studies, there is a growing imperative to develop innovative methodologies that can enhance the current reprogramming paradigm in cardiac cells. In a recent study, our team explored the *Sall* gene family member Sall4 due to its unique roles in pluripotent stem cells, cardiac progenitors, and heart morphogenesis [88]. Our work expands upon the promising findings that overexpressing Sall4 alongside Gata4, two transcription factors known for their distinct and overlapping roles in resident CPCs, iCM transdifferentiation, iPSC and iCPC reprogramming, and heart development, can potently induce partial cellular fate transitions in cardiac fibroblasts. Specifically, the data demonstrated that the overexpression of these two factors drove the emergence of a stem/progenitor-like cell population in rodent cardiac fibroblasts, with approximately 32 ± 6.4% expressing Nkx2.5 and 13 ± 3.6% expressing Oct4. Additionally, 29.2 ± 15.0% were detected as Flk1^+^, while the percentages of Nanog^+^ or SSEA1^+^ cells remained below 5%. These cells exhibited remarkable clonogenicity and extended ex vivo expandability, showing high susceptibility to differentiate into various cardiac and non-cardiac cell types, including contractile cardiomyocytes, endothelial cells, smooth muscle cells, and neuron-like cells. A molecular analysis revealed that the SALL4-GATA4 complex activates crucial stemness-related signaling pathways such as PI3K/Akt, Hippo, and Wnt, while synergistically stimulating pluripotency and cardiac gene promoters and repressing fibrogenic genes. This synergistic action suggests a primitive transition process capable of driving cardiac regeneration. Furthermore, significant stem/progenitor-like transitions and robust cardiogenic differentiation were observed in both human and rodent cardiac fibroblasts but not in skin fibroblasts. This distinction potentially highlights the cardiac specificity and therapeutic applications of this approach. Together, these findings open new avenues for advancing cardiac regenerative therapies and tissue engineering.

**Figure 1:**
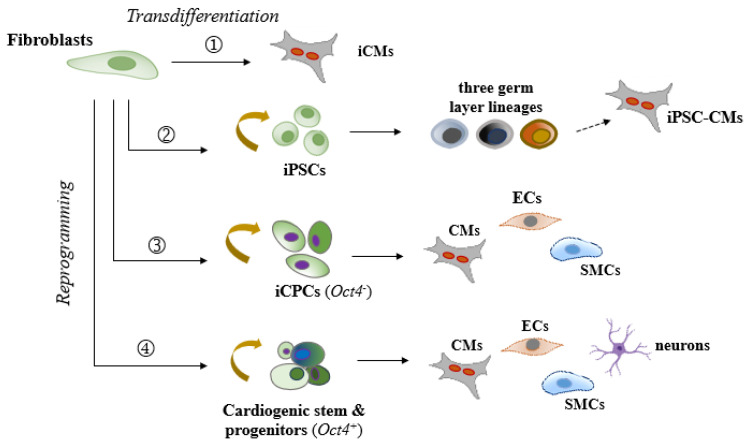
A schematic diagram of current reprogramming methods to regenerate cardiac myocytes and supportive cells from fibroblasts (preferably of cardiac origin) through various cell fate transition states. (1) Direct transdifferentiation into induced cardiomyocyte-like cells (iCMs) solely. (2) Reprogramming into induced pluripotent stem cells (iPSCs), followed by differentiation into tri-germ layer lineages, and then further into iCMs and other desired cell types. (3) Reprogramming into induced cardiac progenitor-like cells (iCPCs), followed by differentiation into the three cardiac lineages: cardiomyocytes (CMs), endothelial cells (ECs), and smooth muscle cells (SMCs). This process typically does not involve the reactivation of key pluripotency factors like Oct4. (4) Reprogramming into a partially cardiogenic stem/progenitor-like state followed by regeneration of cardiac lineages and other reparative cell types, such as neurons. This process may involve key pluripotency factors like Oct4 at variable levels.

In comparison to existing cardiac reprogramming strategies (see Figure 1), this approach presents several unique strengths: (1) the use of only two factors, Sall4 and Gata4, both of which naturally contribute to cardiogenic regeneration; (2) a simplified, cardiac fibroblast-targeted procedure that does not require specialized induction media to drive the process; (3) a high primitive fate (e.g., *OCT4*^+^/*Nkx2.5*^+^) conversion rate in both rodent and human models, along with increased sensitivity to generate multiple heart cell fractions necessary for cardiac repair, which contrasts with the generally low efficiency observed in human iCM reprogramming; (4) anticipated improved myocardial regeneration and integration following localized delivery of these factors; (5) minimal tumorigenic risks in the adult heart, partly supported by in vivo reports utilizing xenotransplantation models or intramyocardial gene delivery in myocardial infarction animal models [89,90]; and (6) given the simplicity of using just two genes, practical non-integrating delivery approaches, such as modified RNA, refined proteins, or CRISPR/Cas9-mediated gene activation technology, that could be readily explored for enhanced safety and efficiency. These strengths make this *non-OSKM* approach highly promising for streamlined therapeutic applications. Nevertheless, despite the compelling advantages, significant limitations need to be addressed in the next steps of the studies. These include (1) a limited understanding of the cellular and epigenetic mechanisms regulating Sall4 and Gata4 interactions in cardiac fibroblasts and fibroblasts from various tissue origins and species, necessitating further investigation to optimize safety, efficacy, and specificity; (2) uncertainty regarding the exact responsive subpopulations within the phenotypically heterogeneous resident cardiac fibroblasts and specific molecular pathways involved, which requires further characterization; (3) challenges in controlling the compositions and fates of reprogrammed and spontaneously differentiated cells, which may affect the consistency and effectiveness of the therapy; (4) the need for systematic in vivo mechanistic and reparative validations to confirm therapeutic benefits in diseased models; and (5) uncertainty surrounding the long-term effects of Sall4/Gata4-induced reprogramming, including safety and sustained functionality in vivo. Taken together, while the potential of Sall4 and Gata4 in cardiac reprogramming is evident, ongoing research is required to address these aspects and fully realize their therapeutic potential.

## 5. Prospective Considerations for Enhancing Therapeutic Cardiac Reprogramming

As the field of cardiac reprogramming progresses, several prospective enhancements warrant consideration to optimize outcomes and broaden therapeutic applicability. Firstly, the choice of starting cells should ideally focus on those of cardiac origin, such as cardiac fibroblasts, which are more applicable for in situ conversion. Utilizing these cells leverages their inherent properties and profiles, as they possess unique epigenetic landscapes and signaling pathways tailored to the heart’s microenvironment. These cells also naturally express many cardiogenic genes that are critical for reprogramming, such as Gata4, Tbx20, Mef2c, and Nkx2-5, which are often absent in non-cardiac cells [91,92,93]. Additionally, cardiac fibroblasts critically engage in scar formation and pathological remodeling following myocardial injury.

Importantly, off-target reprogramming effects can lead to significant undesired consequences. In this regard, distinguishing cell heterogeneity and responsive subfractions through techniques such as single-cell RNA sequencing, phenotypic profiling, and epigenomic analysis will be beneficial for significantly improving efficacy, guiding targeted therapies, and optimizing reprogramming methods. For example, previous studies have identified adult cardiac-resident colony-forming unit-fibroblasts (CFU-Fs) expressing Oct4, but not Sox2 or Nkx2.5 [94]. Diverse progenitor-like fractions have also been characterized based on markers such as Sca1, Isl1, Pdgfrα, Flk1, and Thy1 [38,94,95,96,97]. Such cell subtypes may be more susceptible to specific reprogramming factors. As an example, exogenous Oct4 with either Sox2 or Klf4 partially reprogramed Thy1^+^/Sca1^+^ fibroblasts to a primitive stage concurrently expressing mixed lineage markers [98]. Therefore, targeting cardiac-specific cell types and subfractions ensures relevant and more effective therapeutic outcomes, including the regeneration of lost cardiomyocytes and essential supporting cells through pathways such as paracrine signaling, extracellular matrix remodeling, neurovascular coupling, and angiogenesis—all of which can seamlessly integrate with the heart’s structural and functional demands [99,100,101,102,103].

Secondly, it will be essential to optimize the reprogramming factors and treatment duration, as these elements significantly influence reprogramming efficiency and the resultant cell fate transitions. The Sall4/Gata4 transcriptome study identifies several pluripotency genes and a range of potential transcriptional candidates such as *Sox17*, *Gata6*, *Sall1*, *Tbx1*, and *Srf*, along with other relevant genes like *Hcn4*, which play critical roles in various aspects of the cellular reprogramming process. Briefly, Sox17 and Gata6 are key regulators of endodermal and mesodermal lineage specification, with the interaction between SOX17 and OCT4 being particularly important in cardiac progenitor cell development [104]. Gata6 shares overlapping roles with Gata4 in regulating the expression of Sall4 and inducing pluripotency [105]. Sall1, similar to Sall4, is involved in maintaining pluripotency and dedifferentiation, supporting the transition of differentiated cells back to a more stem-like state while sustaining the cardiac precursor cell-like features [106]. Tbx1 contributes to cellular proliferation, differentiation, and early cardiac development, playing a role in both myocardial growth and angiogenesis [107]. Likewise, serum response factor (SRF) is crucial in regulating the plasticity and adaptability of cardiac fibroblasts [106,108]. Meanwhile, Hcn4 is crucial for regulating the electrophysiological properties of cardiac precursor cells, especially in maintaining pacemaker activity [109,110]. Together, these factors may differentially enhance the potential of partial reprogramming strategies by driving the dedifferentiation and specification necessary for efficient cardiac regeneration. Additionally, a detailed time-dependent investigation of reprogramming will be beneficial for understanding the precise dedifferentiation and cardiac specification phases, ultimately improving the efficacy of partial cardiac regenerative strategies.

Thirdly, logistical constraints in practical applications, such as factor delivery and precise reprogramming control, need careful consideration. Viral methods, like intra-myocardial (IM) injection or catheter-based adenovirus delivery, provide localized transient expression with reduced risks of insertional mutagenesis, though concerns remain regarding immunogenicity and scalability [111,112]. Non-integrating approaches, including small molecules, chemicals, and microRNAs, offer a transient and reversible reprogramming process with diminished risks of long-term genomic alterations. Non-viral delivery methods, such as lipid nanoparticles and cell-penetrating peptides (CPPs), further enhance safety and efficiency. Lipid nanoparticles encapsulate reprogramming factor proteins or mRNAs, protecting them from degradation while ensuring efficient cellular uptake. CPPs enable the direct delivery of reprogramming proteins into cells by crossing the plasma membrane and targeting the nucleus to modulate target gene expression [113,114]. Additionally, CRISPR-Cas9-based transactivation offers further control over gene expression in vivo, enhancing reprogramming accuracy [115]. Leveraging available datasets for these factors, in conjunction with in-depth Sall4/Gata4 epigenetic and microRNA studies, may aid in predicting and establishing new essential effectors. Small molecules can modulate key DNA methylation and histone modification states, as well as signaling pathways such as PI3K/Akt, Hippo, TGF-β, Wnt, and MAPK, all identified in Sall4/Gata4-stimulated pathways in cardiac fibroblasts. Similarly, microRNAs can fine-tune the balance between cellular processes, such as proliferation, survival, and cardiac fate induction, as mediated by Sall4/Gata4. For example, miR-1, miR-133, miR-208, miR-200 family, and miR-590 have been shown to promote cardiomyocyte conversion and phenotypes, as associated to Gata4 or Sall4 [32,116,117,118]. All of these could be harnessed to enhance partial cardiac reprogramming. Additionally, combining reprogramming with tissue engineering strategies, such as 3D bioprinting of cardiac patches or biomaterials [119,120], could offer synergistic benefits by providing structural support and facilitating the integration of newly generated cardiomyocytes into heart tissue.

Lastly, new strategies should emphasize the development of human-specific reprogramming factors and techniques, ensuring that approaches are tailored to the unique characteristics of human cardiac cells. This focus can enhance the efficacy and safety of reprogramming methodologies, ultimately facilitating their translation into clinical practice. Overall, these prospective enhancements—from optimizing cell selection and reprogramming factors to refining non-integrating reprogramming methods and advancing the development of human-specific protocols—represent pivotal steps in advancing cardiac reprogramming therapies toward future clinical applications.

## 6. Conclusions

In conclusion, cardiac partial cell fate transition strategies offer significant potential for addressing the challenges of myocardial regeneration. These approaches leverage the plasticity of iPSCs, promoting broad tissue regeneration and rejuvenation in the diseased heart, while maintaining the lineage-specific safety and reduced tumor risk associated with iCPCs (see Figure 1). By facilitating versatile in situ myocardial regeneration, partial reprogramming strikes a balance between regenerative potential and therapeutic safety. Although significant limitations remain, such as the need for a deeper understanding of intrinsic molecular and epigenetic mechanisms, cellular interactions, and optimization of targeted reprogramming, future advancements are anticipated to bring us closer to achieving safe, effective, and clinically relevant cardiac reprogramming therapies for regenerative medicine.
